# Navigating professional identities: nursing faculty as embedded simulation participants in medical student simulations

**DOI:** 10.1186/s41077-025-00353-3

**Published:** 2025-05-14

**Authors:** Shelley Walker, Eve Purdy, Helen Houghton, William Dace, Victoria Brazil

**Affiliations:** 1 Faculty of Health Science and Medicine, Bond University, Gold Coast, QLD 4226 Australia; 2 Translational Simulation Collaborative, Faculty of Health Science and Medicine, Bond University, Gold Coast, QLD 4226 Australia; 3 Department of Emergency Medicine, Gold Coast Hospital and Health Service, 1 Hospital Blvd, Gold Coast, QLD 4225 Australia; 4 Department of Anaesthesia, Royal Glamorgan Hospital, Ynysmaerdy, Llantrisant CF72 8XR Wales

**Keywords:** Simulation, Interprofessional, Embedded simulation participant, Education, Phenomenology, Professional identity

## Abstract

**Background:**

Nursing trained faculty often work as embedded simulated participants (ESPs) in interprofessional simulations. Blending and switching their professional identities as educators, nurses, and role players in ESP roles can be challenging. How they balance tensions in their role portrayal is poorly understood. New and experienced faculty may benefit from clearer guidance about how to approach this task.

**Methods:**

Using a descriptive phenomenological approach, we explored the experience of nurses working as ESPs in a medical student simulation-based education program. We were sensitised by Dace’s “blended boundaries” model of professional identity for simulation educators. We performed 9 semi-structured interviews with nurses who work as ESPs in our simulation program and undertook a thematic analysis of the transcribed data employing Braun and Clarke’s (2006) six phase approach.

**Results:**

We identified five themes: (1) role complexity, (2) influences and tensions in role portrayal, (3) judgement and flexibility, (4) perceived interprofessional outcomes, and (5) personal and professional impacts.

**Discussion:**

Role portrayal of ESP nurses, by nurses, in interprofessional simulations is a complex and nuanced task. Carefully planned and reflected upon role portrayal offers powerful opportunities for medical students to gain a deeper understanding of interprofessional healthcare teamwork and the unique role of nurses in those teams. Thoughtful role portrayal supports highly authentic scenario delivery and clinical learning outcomes and can have positive professional impacts for the nurses undertaking this role. We suggest simulation programs should be highly intentional when recruiting, training, and supporting nurses to work as faculty in interprofessional simulations.

**Supplementary Information:**

The online version contains supplementary material available at 10.1186/s41077-025-00353-3.

## Background

Nursing trained faculty often work as ESPs in interprofessional simulations, but little is known about their approach to this role. As ESPs, nurses may draw upon multiple roles/identities and may experience tensions between these roles, e.g. ‘holding back’ in portraying professional nursing expertise to allow junior learners to work through a scenario, or ‘being difficult’ to present learners with interprofessional teamwork challenges. Without clearer understanding of how these roles are approached, we may miss opportunities or risk harms within the interprofessional learning experience. Improved understanding will allow clearer guidance for newer faculty members to consider when portraying nursing roles in interprofessional simulation-based education (IPSE).

The term ESP is defined as ‘An individual who is trained or scripted to play a role in a simulation encounter in order to guide the scenario, and may be known or unknown to the participants; guidance may be positive or negative, or a distractor based on the objectives, level of the participants, and the needs of the scenario’ [1]. These roles have been played by specialised simulated participant educators, actors, faculty members, learners or clinicians [2, 3]. The role can vary from a simple script designed to lend authenticity, to a primary educational role as an in-simulation coach or guide [3, 4]. There is extensive guidance for simulated participant (SP) methodology applied to role portrayal of patients/healthcare consumers [3–5]. However, the approach to role portrayal of health professionals by ESPs is both complex and underexplored. It is potentially problematic, for example asking an accomplished professional to portray a more junior or less experienced version of their usual clinical role [3], but if done carefully may powerfully support interprofessional learning outcomes.

Professional identity guides behaviour within professional roles, and we expect this is also likely when assuming the ESP role. The formation of professional identity is a complex process that evolves throughout one’s career. Professional identity will inevitably influence ESP role portrayal by clinicians working in healthcare simulation. Dace et al. [6] described ‘wearing hats’ and ‘blending boundaries’ when simulation faculty draw upon their clinician and educator professional identities. These ‘leaky’, blended boundaries can be helpful or problematic and require flexibility and careful judgement by simulation educators.

The aim of our research is to explore the experience of nursing faculty working in the role of an ESP for our simulation program, with a view to improving our support and guidance for their work.

## Methods

We explored the experience of clinical nurses working as ESPs in a medical student simulation-based education program, using a descriptive phenomenology approach. We were sensitised by Dace’s ‘wearing hats and blended boundaries’ model of combining professional identities as clinicians and educators within simulation-based education in our exploration [6]. For our study the term ‘ESP’ refers solely to the nursing cohort embedded as faculty in simulation scenarios and ‘SP’ to those who portray patients or family members. We work with SPs to portray patients and family members within the medical program where this study took place, but this group is not the focus of our study.

### Study setting

The following section will describe our simulation program in detail to provide context for our study, following published reporting guidelines for healthcare simulation research [7].

The Bond University Medical Program is a 5 year degree, with a ‘pre-clinical’ phase one followed by clinical rotations within health services in phase two. Simulation based education is introduced in the third year, with the following structure:

- Phase one
  o Two simulation sessions in Year 3 with a broad range of clinical cases, e.g. patients with delirium, chest pain, abdominal pain, post operative care, shortness of breath.
- Phase two
  o Peer assisted learning simulation [8] during the general medicine rotation in Year 4
  o Women’s health simulation during the obstetrics and gynaecology rotation in Year 4
  o ‘Deteriorating patient’ simulations during the critical care and orthopaedics rotation, using a ‘Live, Die, Repeat’ format [9] in Year 5
  o Simulated emergency department during which students complete 2 h shifts and interact with multiple SPs [10] in Year 5

The design, delivery, and debriefing of the simulation program is performed by a faculty team of clinician educators (doctors and nurses) who facilitate sessions and trained SPs representing patients or family members. The program places a high value on interprofessional learning outcomes in simulation activities. Qualified nurses working in the ESP nursing role provide a combination of authenticity and experience in interprofessional teamwork for our student participants. The nurses are employed as part of the simulation team. Nurses are recruited for the simulation program via word of mouth by those already employed in the Bond medical program. Hence, they come with prior experience in education and simulation, and good knowledge of the medical program curriculum. Training has been informal, including ‘buddying’ or observing the nurse ESP role in simulations, with guidance or feedback given by more experienced faculty.

For each session, faculty are emailed session details and scenario case files in advance. A faculty pre-briefing is conducted prior to students arriving, to orientate the team to the year level of participating students, clarify learning outcomes, and discuss expectations of simulation delivery and role portrayal.

A typical simulation session has up to six students in each group, participating across three different scenarios. The setting is either in an emergency department or on a hospital ward. The session begins with introductions and an ‘ice breaker’ question to facilitate interaction, followed by pre-briefing. The pre-brief involves clarification of the simulation process, expectations and goals and a brief description of the debriefing process that will follow. Two students then participate in a scenario that includes a nurse ESP and an SP in the role of the patient or relative. In the critical care and Emergency Department simulations, a manikin is used for specific scenarios not suited to an SP. The ESP, in the role of a bedside nurse, provides clinical realism and acts as a guide and prompts when students are struggling or not moving through the scenario as expected. They can also provide role modelling in terms of communication and interaction with the SP. The scenario runs for 8–10 min and concludes with the students giving a handover to a senior clinician, who is usually one of the medical facilitators. Of note, these medical facilitators are also serving as ‘doctor ESPs’ whilst within the scenario, but their experience was not explored in this study.

Each scenario is followed by a debriefing session with the students, led by the medical facilitator. The ESP and SP are included in debrief and are invited to provide feedback/comments for the students. At the conclusion of the session, students are invited to share something that they learned from the experience.

### Data collection

Nursing faculty working in the Bond medical student simulation program as ESPs were invited to participate in the study via email. Nurses were also verbally advised of the research project during simulation sessions prior to recruitment. Given the small size of the ESP program, we attempted to schedule an interview with any nurse who wished to participate.

Data was collected via interviews using semi-structured and explicitation techniques, with an interview guide that was developed EP and SW. The semi-structured questions were designed to gather information on experience and role perception, whereas the explication questions took a deeper dive into the ESP experience by asking participants to reflect closely on aspects of recent simulation sessions. A similar approach is described by Bedin et al. in their study of older adults [11]. The interview script is in Appendix 1. Interviews were recorded and transcribed (otter.ai), then reviewed for transcription accuracy by the interviewer. The interviews were performed by E.P. and S.W. Interviewees were given the opportunity to review their transcript prior to de-identification and analysis.

### Data analysis

Data collected from interviews was analysed using thematic analysis, employing Braun and Clarke’s (2006) six phase approach [12]. All authors initially familiarised themselves with the data from all interviews. They then each performed a more detailed review of 3 transcripts independently, identifying codes within the data. This was followed by a group meeting to compare analyses and generate a draft of themes and subthemes, which was then reviewed and refined by E.P. and V.B. At this point we collectively reflected on whether there were ongoing questions that needed further data—either by returning to participants who had already participated or recruiting new participants that had not volunteered in the initial round of interviews. Using these draft themes, the author group reviewed the transcripts again, to identify any new concepts and select illustrative quotes for each theme. Transcripts were then reviewed a final time by S.W.

Ethical approval was granted by the Bond University Human Research Ethics Committee, reference number SW02111.

### Reflexivity

The research team composition represents a variety of perspectives and shared experience of combining clinician, educator and simulation roles. S.W. is an emergency physician and current assistant professor in the Bond medical student simulation program. H.H. is a nurse and midwife, and lead simulation program coordinator whose responsibilities include recruitment, rostering and support for nurses working in the simulation program. V.B. was previously the director of the Bond medical student simulation program and continues to work as one of the medical facilitators. She has over 20 years’ experience in simulation education and research, and is an emergency physician. E.P. is an emergency physician and anthropologist, with extensive experience in qualitative research, including in the simulation context. W.D. is a trainee anaesthetist who previously worked as a medical facilitator within the Bond program. He is the first author of the ‘blending boundaries’ article [6] that sensitised our research question and data interpretation.

Throughout the project we have reflected on our positioning related to this work. The embedded nature of our roles within the Bond simulation program gives us rich insights and allows for the rapid ability to achieve depth in interviews, but may also limit the interpretation of findings in some ways due to pre-existing notions. We attempted to mitigate some hierarchical realities by having E.P. and S.W. (who do not have a leadership role in the program) conduct the interviews. We feel the benefits of insider status during the interviews outweighed the potential drawbacks for the type and depth of the experience we were trying to make sense of. Our findings are the product of the interplay between the data and the perspectives we bring from these roles.

## Results

### Study participation

Of the eighteen nurses that are employed as ESPs at Bond University, nine (50%) participated in initial interviews. The duration of experience within the Bond simulation program ranged from less than six months to more than ten years. Most participants had been ESPs for 2–8 years. Following review of the nine interviews the research team decided not to perform further interviews given the commonality of themes identified and the low likelihood of identifying other significant findings.

### Themes

We identified five themes in the data: 1) Role complexity, 2) Influences and tensions in role portrayal, 3) Judgement and flexibility, 4) Perceived interprofessional outcomes and 5) Personal and professional impacts. The themes will be elaborated in the following sections, with illustrative quotes attributed by study participant number (e.g. [N1]).

#### Theme 1: Role complexity of the ESP

Our primary finding was that the nursing ESP role is both critical to the success of the simulation and a challenging job to do well. Superficially the role could be seen as a simple realism prop, just ‘*being your everyday nurse’* [N7]. However, there was also awareness of the educator role and the need to model desired behaviours. Other roles identified included managing scenario flow, fostering psychological safety, and keeping simulated patients safe.

Promoting realism within the simulation scenario contributes to student engagement and helps provide an immersive experience. Nursing ESPs described deliberate strategies for promoting realism such as actively demonstrating engagement with SPs as they would with a patient. In some cases, they felt immersed in the scenario themselves, especially during the Emergency Department simulation:

So I pretty much just went into work mode and even just trying to get the students to think about disposition. It’s things I would do normally so it was just honestly, I felt like I was at work. [N1]

Nursing ESPs described the importance of fostering psychological safety during scenarios and an approach to help create conditions where students could take risks and be supported. They also recognised their role as an educator both through role modelling and through more direct means. They consistently highlighted the opportunity to role model good clinical behaviour and communication as a part of their role within the scenario:

I feel like part of the nursing role is to role model professionalism, good teamwork, communication. [N8]

Our study participants described being sensitive to the SP and their safety. Examples of this include checking in on SP comfort, both in their role and generally.

Professionalism, so like making sure that the students interacted in an appropriate manner with the simulated patients, actually do take it seriously. [N4]

Nursing ESPs considered themselves as educators, with a range of responses regarding how they conceptualised that role. The most common descriptors were guide or resource, with nursing ESPs describing methods to encourage students to think for themselves and access prior knowledge.

I feel like if I just give them the answer, they won't retain it yet if they actually have to sit there and you know, feel a bit uncomfortable not knowing the answer and working it out. They're gonna have far better results and retention of that answer. [N4]

#### Theme 2: Influences and tensions in role portrayal

Several influences were identified by nursing ESPs in their choices regarding role portrayal, including scenario learning objectives, simulation team de-briefing, peer observation, coaching/workshops and informal feedback. Individual learner needs also influenced role portrayal during scenarios.

Nursing ESPs used information emailed prior to simulation sessions as a trigger to refresh knowledge in preparation for the session based on the scenarios and learning objectives:

I was trying to refresh my knowledge on something or if there's going to be medications involved that I may not be that familiar with or something like that. So definitely always pre read and I like to be organized. [N1]

Faculty pre-briefing sessions were a valuable guide that influenced nursing ESP role portrayal for that session. Overall, nursing ESPs described being focused on student learner experience and paying close attention to understand how best to help the students throughout the simulation.

You’ve just got to listen to what the student needs, just like your patients. Is there anything else I can help you with? It's not about me having an agenda that they need to do six things properly. [N3]

The influence of professional identity on role portrayal was found to be highly complex. Some nursing ESPs described apprehension around teaching in a ‘medical’ space. In contrast, many of our nursing ESPs expressed initially feeling as if they were misrepresenting their profession. Not taking the initiative that a senior nursing professional would usually take in a clinical encounter was especially jarring for some. However, this perception shifted with experience—student learning became a stronger focus for them rather than what they would usually do in the clinical space. The concept of ego within professional identity was seen as something that needed to be put aside to be a better educator.

Because in the beginning, I really wanted to say, I'm not really that dumb. You know, I'm not really that dumb nurse or, you know, I wanted to be able to say yeah, I do want to be able to say those things. And so I would find it really, I had to be very intentional to let that go and just go it's okay. This is for their learning. This is not for me to feel good. And yeah, just let that role play out. [N2]

#### Theme 3: Judgement and flexibility to navigate blended boundaries in the ESP role

Navigating the blended boundaries of nurse/educator as an ESP requires a significant amount of judgement and flexibility from session to session, scenario to scenario, and learner to learner. This is a high-level, dynamic cognitive and social process that our nursing ESPs go through during each scenario.

And again, you just come in and out of role depending on what you see them do, as well. So you've got to really concentrate and listen and watch what they're doing. And then step in, if they need help, and perhaps change to an educator, just a little bit. So obviously, as you get more experienced, you'll learn to be quiet, maybe give them a sentence give them a clue try and look at things, how else can I help them transition at this particular time and be very patient and kind. [N3]

Management of scenario flow and pace, ‘*to keep the flow going, to make sure that they (students) were talking to the patient in the right direction*’ [N7], was a subtle and highly nuanced task described by nursing ESPs. They saw their educator role as particularly important when students were 'stuck' and used techniques such as offering investigation results as a way of moving students forward. This sometimes presented a challenge—how much scenario flow management by the nursing ESP is desirable? In some instances, the learning value of being stuck is lost by moving forward or managing flow.

Dace’s [6] ‘hat model’ was discussed during interviews, with some study participants describing having to '*intentionally switch hats*' [N2].

…it's difficult line in a way to walk. Because you don't want to be teaching too much. You want to make it as realistic as possible. So you want to pretend that you're the nurse. Not make it feel to them like you're pretending but just so that they feel that you are a nurse and so responding as you would in that situation, but not perhaps guiding and prompting them too much. But yeah, so that can be a tricky line to walk, but I think that's what makes it challenging and interesting. Kind of putting your actor hat on and your educator hat on and your nurse hat on. [N9]

#### Theme 4: Perceived interprofessional outcomes

Nursing ESPs acknowledged that their role had several interprofessional outcomes, including role modelling, development of trust in other health profession roles and encouragement of help seeking behaviours.

Role modelling clinical encounters and communication was perceived as an opportunity to mirror what should happen in the workplace.

So it’s kind of the one thing I think, Bond does really well from a simulation point of view is focus on team environment and that role play in simulation is important part of that well this should be your goal, your cultural norm. [N6]

Nursing ESPs actively encouraged students to seek help, whether that be references or guidelines, or senior clinician input. They were hopeful that students would be more able to seek help in the workplace and recognise the team environment.

A very positive impact, I think and I think that they find it comforting to know that when they get into the real world there will be somebody who's actually on the same team and happy to help them and not some scary, unknown role. [N9]

#### Theme 5: Personal and professional impacts

Nursing ESPs outlined several personal and professional impacts of being involved in the ESP program. They described an enhanced understanding of medical roles and personal clinical development, which included teamwork and communication with other health professionals.

Yeah so to be able to talk through and unpack some of those learning opportunities even from the experienced nurses. I think the simulations have helped in how to do that, and how to give and receive feedback, because I've definitely learnt a lot in how to do that. [N2]

## Discussion

Our goal was to explore nursing trained faculty’s experience of their role as ESPs in the Bond University medical simulation program to better define and support the role. The findings will inform improvements to our program and may offer insight to other simulation programs who have similar clinical ESP roles. Because every program is different – we feel that a commitment to explore and improve the ESP experience within each local context is critical. Our findings can serve as a starting point.

The results of our study align with current ESP role descriptions in the literature, such as providing guidance, adding realism, and fostering psychological safety [2, 3]. However, we have revealed a much deeper and more layered experience of the nursing ESP role with significant tensions, complex and nuanced decision making when ‘in role’ and positive effects on other clinical roles.

Addressing the tensions uncovered between professional identities—specifically representing the nursing profession well versus facilitating a useful education experience – is a challenge. Feelings of acting like a ‘dumb nurse’ highlighted an unintended (never explicitly requested) and potentially harmful outcome of our current simulation education program. Although this feeling appears to be mitigated by experience, and by recognising and embracing the role of educator, we have reflected on how to better support nursing trained faculty to reconcile this tension.

Likewise, the complex decisions nursing ESPs make throughout a simulation scenario in response to the student learners’ actions has been under recognised. These decisions require careful attention to the students’ actions and level of training, the SP and clinical encounter, and knowledge of the session learning objectives. Providing support, appropriate guidance and meaningful feedback for these decisions may enrich the role and experience of both our ESPs and students.

**Fig. 1:**
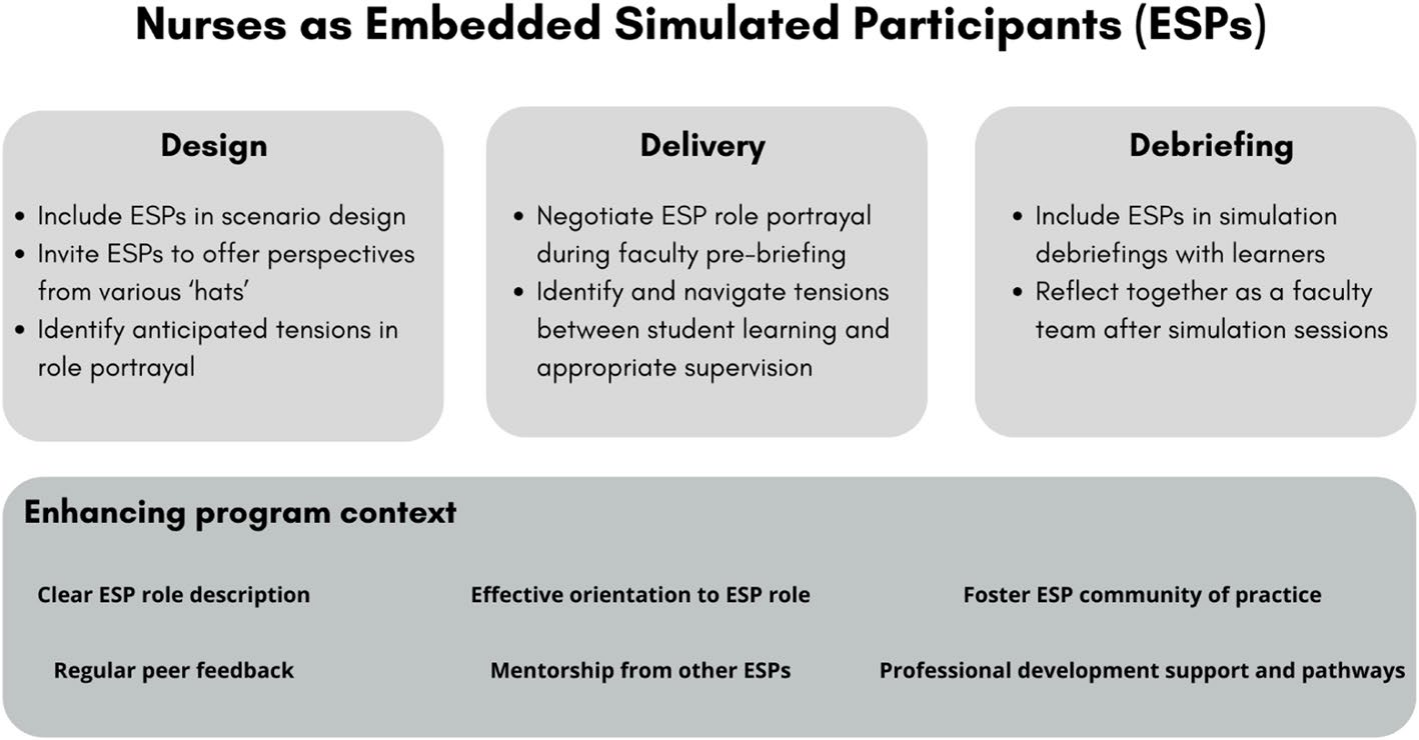
Program considerations

We have reflected on our preparation and support for nurses as ESPs in our simulation program because of this study. We agree with Nestel et al. [3] that a systematic approach to training for role portrayal drawn from SP methodology is a useful starting point for clinicians as ESPs, i.e. preparation for ‘person’, learning activity, context and rehearsal. We plan to create a more formal written role description for the nursing and ESP roles within our simulation program. Cognisant of the tensions uncovered in nursing ESP role portrayal, we are reviewing scenario design elements to identify where these tensions might be felt most acutely, and scheduling a faculty debrief at the end of the simulation session to reflect on the issue. This will further inform the faculty pre-briefing conversations that offer practical and contextual guidance for nursing ESPs just prior to the simulation session delivery. We plan to incorporate explicit discussion of these issues in our faculty professional development activities. Figure 1 includes suggested considerations for each phase of the simulation process and broader simulation program context.

Missing from our findings was reflection on experiences of being asked to portray difficult or unhelpful nurses (e.g. nurses who ‘go to tea’ in inopportune moments) to challenge participants, despite this phenomenon being not uncommon in clinician ‘confederate’ roles [3]. We expect this is because our simulation program has a strong espoused value about avoiding unhelpful stereotypes in simulation, due to the many wider negative outcomes from these practices [13]. However, it was instructive that even this strong message from program leadership did not completely inoculate against potential negative impacts of feeling like (and concerns for looking like) a ‘dumb nurse’.

**Fig. 2:**
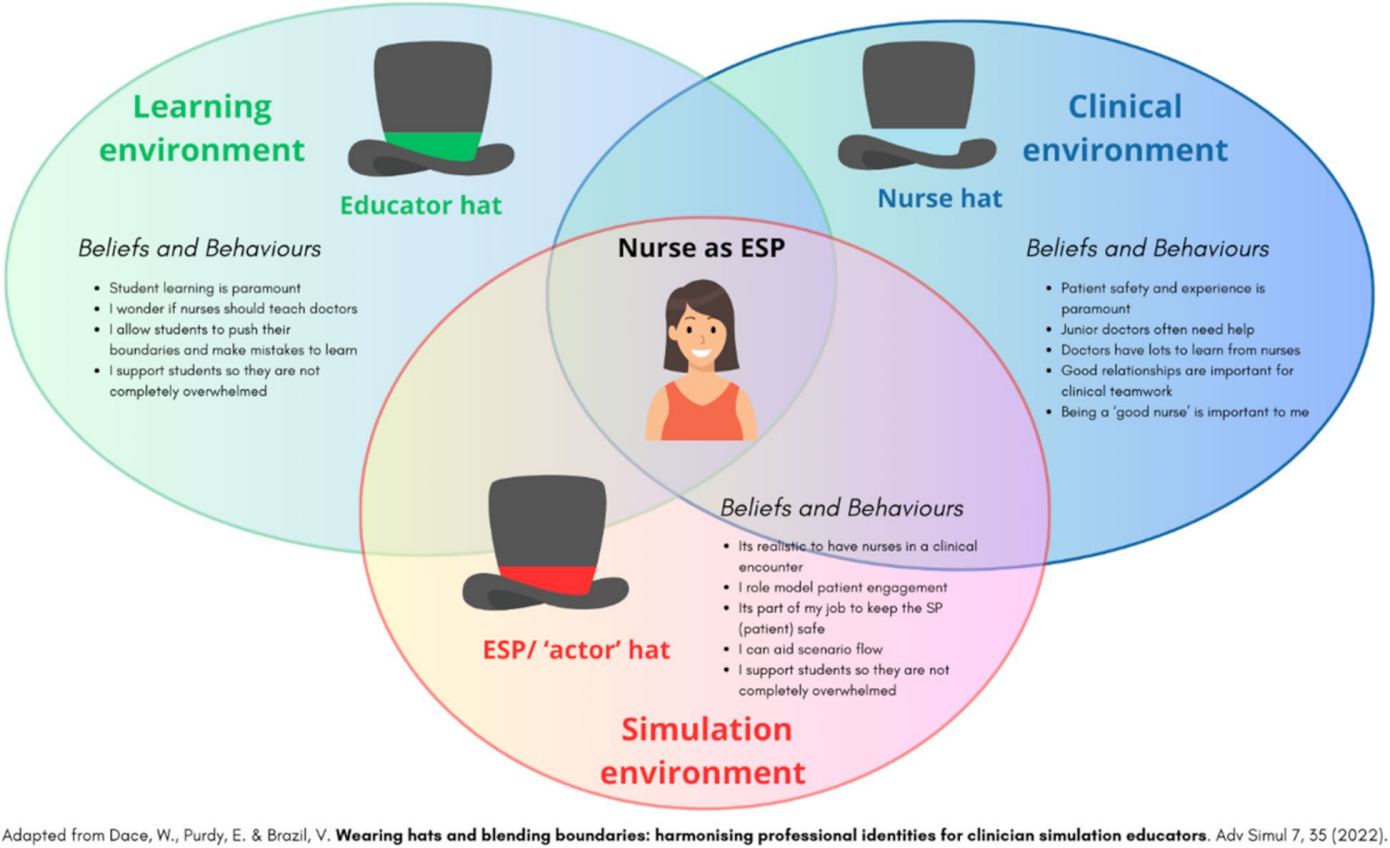
Dace’s hat model applied to nurses as ESPs

Although our study focused specifically on nursing ESP roles, we have reflected that these same tensions and judgements are present for any clinicians undertaking clinician ESP roles. Dace’s [6] model of ‘wearing hats’ and ‘blending boundaries’ appears to be applicable in the context we explored, where the professional identities of nursing faculty as clinicians, educators and professionals were influential and ‘toggled’ during scenario role portrayal. We have illustrated the adaptation of the model to this context in Fig. 2.

## Conclusions

Our exploration of nursing faculty working in ESP roles in our simulation program has uncovered important insights, with implications for our scenario design and delivery, and for preparation and support of nursing faculty who work in ESP roles. The choices made in role portrayal appear to have powerful impacts – on learners, on the nurses working in ESP roles and the values signalled by the simulation program. We hope these insights prove useful for simulation programs that employ clinicians as ESPs.

## Supplementary Information

Additional file 1. Appendix: Nurses as ESPs participant interview.

## Data Availability

The datasets generated and/or analysed during the current study are not publicly available due to potential confidentiality issues but may be available from the corresponding author on reasonable request.

## Abbreviations

ESP: Embedded simulated participant
IPSE: Interprofessional simulation-based education
SP: Simulated participant
