# Supplementary material for: Navigating professional identities: nursing faculty as embedded simulation participants in medical student simulations
Source: Adv Simul (Lond). 2025 May 14;10:28. doi: 10.1186/s41077-025-00353-3 (PMC12079885; doi:10.1186/s41077-025-00353-3)
Supplement: Supplementary file 1 — Additional file 1. Appendix: Nurses as ESPs participant interview. [file 41077_2025_353_MOESM1_ESM.docx]

Nurse Embedded Simulation Participant Interview

This interview contains 2 types of techniques, semi-structured and an explicitation interview.

*Thanks for taking the time to speak with me today. We will talk for about 30 minutes, during which I hope to learn more about your role as a nurse embedded simulation participant. Questions will be about your role in the Bond Medical School simulation program, with a particular emphasis on how you view your role, thoughts on the different ‘hats’ involved eg professional nurse, educator, and how involvement in this program affects other aspects of your professional life. I will record this, it will be transcribed then de-identified prior to being shared with any of the others on the research team. I will share your transcript with you in the next week and you can make any revisions.*

*I want to remind you that what we talk about today will not impact your employment at Bond. I don’t have any say over your employment at Bond. Are there any questions about this before we start?*

Introduction – Semi Structured

- Tell me about your role as a nurse embedded simulation participant...
  - How long have you been doing it?
  - Why did you start?
  - Do you have any prior education experience?
  - How did you learn about your role?
  - What other jobs do you have?
- Tell me about your role as a nurse in the simulations?
- Tell me about your role as an educator in the simulations?
- Describe the most important thing you thinks students learn from you?

Lived Experience – Explicitation Interview

*In this part of the interview I am going to ask you to recount, in as much detail as possible your experience as an embedded simulation participant.*

1. I would like you to think back to the last simulation you participated in. Take me through that experience from preparation through to debriefing.
   1. Any follow up questions have the goal of keeping the participant on track of eliciting this experience or to further explore in depth the experience. They can be related to:
      1. Setting the scene (getting more detail about the event) – *i.e. describe where you were standing, describe the group you were working with*
      2. Obtaining more precise understanding about the experience – *i.e. what were you paying attention to? How did you know that?*
      3. Investigating decision making processes – *i.e. when the participants did xx what did you think? How did you decide when to intervene?*
      4. Understanding the underlying views/values – *i.e. what was most important to you?*
2. I would like you to think back to a simulation that you have participated in where you felt that participants needed a fair bit of support from you as the nurse….Take me through that experience from the beginning of the simulation…
   1. Any follow up questions have the goal of keeping the participant on track of eliciting this experience or to further explore in depth the experience. They can be related to:
      1. Setting the scene (getting more detail about the event) – *i.e. describe where you were standing, describe the group you were working with*
      2. Obtaining more precise understanding about the experience – *i.e. what were you paying attention to? How did you know that?*
      3. Investigating decision making processes – *i.e. when the participants did xx what did you think? How did you decide when to intervene?*
      4. Understanding the underlying views/values – *i.e. what was most important to you?*

Hats – Semi-Structured

*Thanks for sharing those experiences. Now just a few final questions.*

- We have heard some of the nurse embedded simulation participants (or if relevant to experiences above) talk about wearing their “nursing hat” or their “educator hat” at different times in simulation….tell me about the relevance of these different hats to you.
  - How have you learned to navigate these dual identities?
  - How do you know when it is time to “switch”
  - What impact do these roles have on the students you work with?

Impact on Work – Semi-Structured

- How does working as an embedded simulation participant impact your clinical work, if at all? Has it changed how you interact with junior staff members?
- How does working as an embedded simulation participant affect any other educational roles?

*Thanks for your time….Do you think Is there anything else that I have missed?*
